# Total Hip Arthroplasty with the Conservative Cementless MINIMA Size 1 Stem in Patients with a Small Femoral Canal: 3–6 Years of Follow-Up

**DOI:** 10.3390/jcm15020861

**Published:** 2026-01-21

**Authors:** Maros Hrubina, Marian Melisik, Zoltan Cibula, Peter Lisy, Juraj Cabala, Milan Cipkala, Lubica Kasakova, Jana Hrubinova

**Affiliations:** 1 Jessenius Faculty of Medicine in Martin, Comenius University in Bratislava, 036 01 Martin, Slovakia; melisik2@gmail.com (M.M.); peter.lisy@gmail.com (P.L.); 2 University Department of Orthopaedic Surgery, University Hospital Martin, Kollarova 2, 036 59 Martin, Slovakia; juraj.cabala@gmail.com (J.C.); milan.cipkala@gmail.com (M.C.); lubica.manakova@gmail.com (L.K.); 3 Physiotheraphy and Rehabilitation Department, University Hospital Martin, Kollarova 2, 036 59 Martin, Slovakia; jhrubinova@seznam.cz

**Keywords:** hip arthroplasty, cementless fixation, femoral canal, prosthesis design

## Abstract

**Background:** The objective of this study was to evaluate the short-term clinical and radiological outcomes of a conservative cementless stem (Minima) in total hip arthroplasty (THA) for patients presenting with a narrow femoral canal. **Methods:** We retrospectively analyzed 18 patients (18 THAs) who received a size 1 Minima stem between 2018 and 2022. Clinical assessment was performed using the Harris Hip Score (HHS). Radiological evaluation focused on stem migration, trabecular bone development, cortical hypertrophy, and the presence of radiolucent or reactive lines. Implant survival was determined using Kaplan–Meier analysis. **Results:** The mean patient age was 51.6 years, with an average follow-up of 57 months. The mean HHS improved significantly from 38.3 preoperatively to 96.4 at the final evaluation (*p* < 0.001). Initial stem migration occurred in two hips (11.1%) within the first 6 postoperative months, with no further progression or loosening observed thereafter. Bony trabecular development was identified in Gruen zones 3 (27.8%), 4 (5.5%), and 5 (16.7%). Reactive lines were present around four stems (zones 3–5). One intraoperative complication (5.5%) occurred (acetabular component migration during trial reduction), which required screw fixation. No revisions were performed. Both clinical and radiological implant survival at the final follow-up was 100.0%. **Conclusions:** At a mean follow-up of 57 months, the use of the size 1 Minima stem in patients with a narrow femoral canal demonstrated excellent clinical and radiological outcomes. These findings suggest that this conservative stem is a reliable option for this specific patient population.

## 1. Introduction

Cementless total hip arthroplasty (THA) in patients with a narrow femoral canal can be a technically demanding procedure, often complicated by pre-existing anatomical deformities such as developmental dysplasia of the hip (DDH), juvenile rheumatoid arthritis, or achondroplasia [1]. Typical femoral alterations include a stenotic medullary canal, an increased caput-collum-diaphyseal (CCD) angle with metadiaphyseal mismatch, and excessive femoral anteversion [2]. In modern orthopedics, the choice of fixation remains a critical decision; while cemented fixation has historically been the gold standard, there is a global trend towards cementless components, especially in younger and more active patients. Recent epidemiological data and registry-based studies highlight a significant shift in the distribution of hip arthroplasty methods, with cementless fixation becoming the predominant approach in many regions due to its potential for biological integration and long-term stability [3].

Such cases frequently necessitate the use of the smallest available femoral components, presenting a significant surgical challenge and an increased risk of complications due to the limited space for optimal implant positioning [4,5].

Despite its clinical importance, there is a paucity of studies reporting the outcomes of cementless stems specifically in narrow femoral canals [6,7]. Given that the majority of these patients are relatively young, conservative (short) cementless stems represent a compelling treatment option; however, data regarding their performance in this specific population remain limited. Following favorable outcomes with ultra-short cementless stems in DDH patients, our department implemented the use of the Minima short stem in patients with dysplastic hips and narrow femoral canals [8].

Since then, we have utilized the smallest available size (size 1) in a significant cohort of patients. The aim of this study is to provide a clinical and radiological assessment of a patient series undergoing primary THA with the Minima stem in small femoral canals, with a minimum follow-up of three years.

We hypothesized that the short monolithic stem would provide a reliable treatment option for patients with narrow femoral canals in the short-to-midterm period.

## 2. Materials and Methods

### 2.1. Patients and Study Design

Patient records were retrospectively reviewed for demographic data, surgical details, and clinical outcomes. To ensure transparency in our cohort selection, we initially assessed 24 patients with narrow femoral canals treated during the study period. A total of 5 patients were excluded based on the exclusion criteria (3 due to previous femoral osteotomy and 2 due to inflammatory joint disease). Of the remaining 19 patients who met the inclusion criteria, one patient was excluded due to loss to follow-up.

The study cohort comprised 18 patients (18 hips) with stenotic femoral canals who underwent primary total hip arthroplasty using the Minima S stem (size 1) between 2018 and 2022 at our University Department of Orthopedic Surgery.

**Inclusion criteria:** Patients aged >18 years with a narrow femoral canal (Dorr type A or B) [9] and a minimum follow-up of three years.

**Exclusion criteria:** Previous hip surgery (femoral osteotomy, trauma), oncologic or inflammatory diseases of the affected joint, osteoporosis, or incomplete follow-up.

### 2.2. Surgical Technique

Preoperative planning and digital templating were performed to determine optimal implant size. All procedures were conducted in the supine position using an anterolateral approach. Uncemented cups (Delta PF, Delta TT, or Delta ST-C; LimaCorporate, Udine, Italy), ranging from 44 to 52 mm, were implanted with a lateral inclination of 40–45° and 15° of anteversion, followed by ceramic liner insertion. The Minima size 1 trial stem (LimaCorporate, Udine, Italy) was then inserted. Hip stability and limb length discrepancy (LLD) were assessed via trial reduction. The final stem was standard in 17 cases and lateralized in one case. The choice between a standard and a lateralized stem was based on the intraoperative assessment of soft tissue tension and stability of the hip with restoration of femoral offset. The lateralized stem (used in one case) was selected when the standard trial stem failed to provide adequate abductor tension or when the reconstructed offset was more than 5 mm lower than the contralateral side, despite achieving equal leg length. Ceramic heads (32 mm or 36 mm) were selected based on the shell/liner diameter.

### 2.3. Postoperative Protocol

Active and passive mobilization with physiotherapy commenced on the first postoperative day. Patients were allowed to stand on the second postoperative day and were discharged at a mean of five days (range: 4–7 days). Protected weight-bearing with crutches was mandatory for six weeks to prevent early migration or rotational stress. Following clinical and radiological review at six weeks, partial weight-bearing was permitted, progressing to full weight-bearing by the third postoperative month. Clinical and radiological follow-ups were conducted at six months, one year, and annually thereafter, with the final evaluation performed in 2025 (mean follow-up: 57 months).

### 2.4. Clinical and Radiographic Assessment

Clinical outcomes were measured using the Harris Hip Score (HHS) preoperatively and at the final follow-up [10]. Secondary measures included thigh pain and any early or late complications necessitating revision.

**Figure 1:**
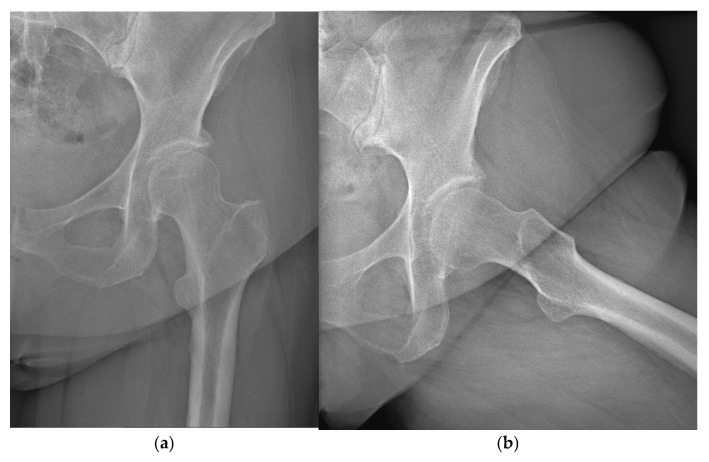
(**a**,**b**): Standard projections—preoperative anteroposterior and axial radiograph of a 58-year-old patient with secondary postdysplastic coxarthrosis of the left hip joint (Crowe type I). The narrow femoral canal (Dorr type A) is presented.

Radiographic evaluation was performed blindly by an experienced orthopedic radiologist using orthogonal projections (anteroposterior and lateral) (Figure 1a,b).

Images were calibrated for magnification based on the known femoral head size.

All radiographic parameters, including LLD, were measured using the measuring tools of the Picture Archiving and Communication System (PACS) digital environment. LLD was defined as the vertical distance between a line tangential to the inferior borders of the ischial tuberosities and the most prominent point of the lesser trochanters.

**Figure 2:**
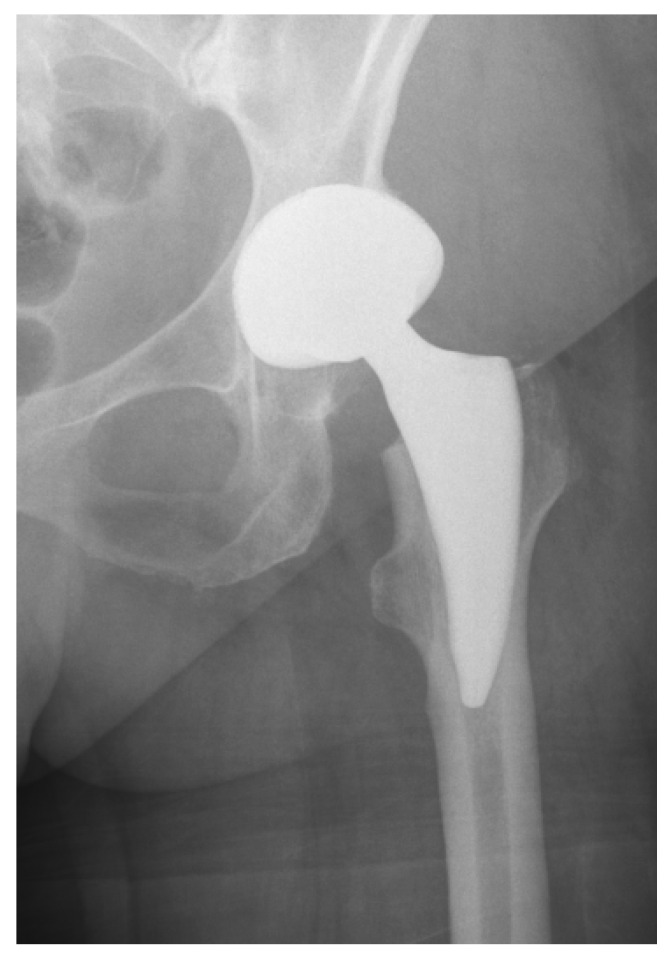
Anteroposterior radiograph of the same patient on the first day after cementless total hip arthroplasty with the Delta TT cup and Minima stem size 1, placed in a neutral position (alignment).

Preoperative parameters included Dorr classification, femoral canal width (2.5 cm below the lesser trochanter), and the caput–collum–diaphyseal angle. Postoperative assessment focused on cup inclination, stem alignment (neutral, varus/valgus) according to Gombar et al. [11] (Figure 2).

**Figure 3:**
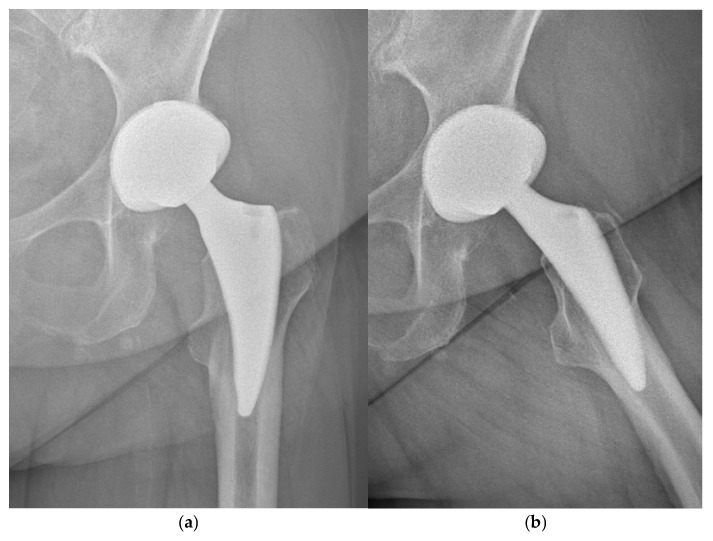
(**a**,**b**): Anteroposterior and axial radiographs of the same patient, 6 years postoperatively, show osseously stable stem fixation with visible femoral cortical hypertrophy in Gruen zones 3–5 (around the distal part of the stem) and a grade I stress-shielding phenomenon in Gruen zone 7.

Radiological evaluation during the follow-up period was focused on: stem migration, the presence of radiolucent lines around the cup and stem, stress-shielding phenomenon, reactive lines, femoral cortical hypertrophy, and bony trabecular development in Gruen zones [12,13,14,15]. Stem migration was assessed according to Martel et al. [16]. Implant stability was categorized as osseously stable, fibrously stable, or loose, per Engh et al. [17]. Loosening was defined as a radiolucent zone >2 mm or migration >3 mm associated with a circumferential radiolucent line [8,11,14]. Stress-shielding and heterotopic ossification were evaluated [18,19] (Figure 3a,b).

A subset of 20 randomly selected radiographs was re-evaluated after two weeks (by L.K. and M.C.) to assess interobserver reliability using intraclass correlation coefficients (ICCs). ICCs ˃ 0.80 were considered excellent.

### 2.5. Statistical Analysis

Statistical analysis was performed using R software (version 4.0.2, Foundation for Statistical Computing, Vienna, Austria). Continuous variables are expressed as mean ± standard deviation (SD). Normality was verified using the Shapiro–Wilk test. Pre- and postoperative HHS values were compared using a two-sided paired Student’s t-test, with significance set at *p* < 0.05. A power analysis indicated that 15 hips were required to detect a large effect size (d = 0.8) with 80% power at alpha 0.05 [20]. Implant survival was estimated using the Kaplan–Meier method.

The data analyses (by performing multivariate regression modeling) were realized: logistic regression for binary outcomes and linear regression for continuous outcomes.

## 3. Results

### 3.1. Clinical Analysis

All 18 patients (18 hips) were followed up for a mean duration of 57.2 months (range: 37–77 months; SD: 13.3 months). The primary indications for total hip arthroplasty were hip dysplasia (*n* = 13), osteoarthritis (*n* = 4), and avascular necrosis of the femoral head (*n* = 1). All procedures were unilateral and performed exclusively in female patients. At the time of surgery, the mean age was 51.6 years (range: 24–71 years; SD: 11.5 years), and the mean Body Mass Index (BMI) was 24.8 kg/m^2^ (range: 19.05–32.83 kg/m^2^; SD: 3.8 kg/m^2^). The mean preoperative HHS improved significantly from 38.3 (range: 24–56; SD: 6.1) to 96.4 (range: 84–100; SD: 4.2) at the final follow-up (*p* = 0.001). Excellent scores (90–100 points) were achieved in 14 patients (77.8%), while 4 patients (22.2%) had good scores (80–89 points); no fair or poor scores were recorded. The preoperative LLD improved from a mean shortening of 0.8 cm to a mean elongation of 0.0 cm (range: 0.0–0.5 cm; SD: 0.2 cm), representing a statistically significant correction (*p* < 0.01) (Appendix A).

Mild thigh pain was reported by one patient (5.6%) who presented with a fibrously stable stem.

### 3.2. Radiographic Outcomes

Preoperative femoral morphology was categorized as Dorr type A in 12 hips (66.7%) and type B in 6 hips (33.3%). The mean width of the femoral canal was 8.6 mm (range: 7–13 mm; SD: 1.9 mm). The mean CCD angle changed from 137.0° (range: 116–153°; SD: 7.7°) preoperatively to 135.0° (range: 134–146°; SD: 2.9°) postoperatively.

The mean initial cup inclination was 44° (range: 40–56°; SD: 5.1°). No cup migration was observed. A non-progressive, 2 mm radiolucent line in DeLee–Charnley zone II was detected in one asymptomatic cup at 3 months postoperatively and remained stable at the 6-year follow-up.

Postoperative stem alignment was neutral in 16 hips (88.9%) and varus (up to 10°) in two hips (11.1%). According to the Engh criteria, 17 stems (94.4%) were osseously stable, and one (5.6%) was fibrously stable; no stems were loose. The fibrously stable stem exhibited initial subsidence (<2 mm) and varisation (<10°) within the first 6 months, with no further progression.

Radiological findings included reactive lines in four stems (22.2%, Gruen zones 3–5), femoral cortical hypertrophy in two hips (11.1%, Gruen zones 2–5), and Grade I stress-shielding in five stems (27.8%, Gruen zone 7). Bony trabecular development was identified in five osseously stable stems, primarily in Gruen zone 3 (27.8%), zone 4 (5.6%), and zone 5 (16.7%). Heterotopic ossification (Brooker type I) occurred in two hips (11.1%) (Table 1).

### 3.3. Complications and Survival Analysis

Complications occurred in two hips (11.1%) without necessitating revision. One intraoperative acetabular component instability occurred during trial reduction, requiring re-reaming and screw fixation; at the 6-year follow-up, the patient was asymptomatic with stable implants and without the signs of ceramic insert destruction. A ceramic insert was selected based on local anatomical conditions. Screw heads remained safely below the internal shell surface, and patient age was considered during the decision-making process.

In a second case (7 mm canal width), the canal required drilling prior to rasping; at 5 years, the patient was pain-free with an osseously stable stem.

Kaplan–Meier survival analysis demonstrated a 100.0% clinical and radiological survival rate at a mean follow-up of 57 months, with no cases of revision for aseptic loosening or any other cause.

Age and gender were not significant predictors in any of the regression models.

## 4. Discussion

The clinical and radiological outcomes of conservative cementless short-stem total hip arthroplasty in patients with primary osteoarthritis, developmental dysplasia of the hip, or femoral head osteonecrosis have been extensively documented [2,8,11,13,14]. Conventional contraindications for short stems include severe dysplasia (Crowe types III and IV) associated with excessive femoral neck anteversion and Dorr type C femoral canals [2,14]. Short conservative stems were developed for younger populations to alleviate thigh pain, preserve bone stock for potential future revisions, and minimize periprosthetic bone resorption [11,13]. However, potential disadvantages reported in the literature include stem malpositioning in narrow medullary canals and undersizing, which may compromise initial stability and lead to fibrous fixation or aseptic loosening [21]. Building on our favorable experience with the ultra-short Proxima stem, we extended this philosophy to the Minima stem in borderline clinical scenarios involving stenotic femoral canals.

The Minima stem (Class 3A) features a triple-tapered, metaphyseally stabilized design. Its porous titanium surface (200 µm pore size) in the metaphyseal region promotes osseointegration, while the roughened distal surface facilitates bone ongrowth [15]. The squared cross-section provides initial torsional stability, and the triple-tapered geometry is designed to prevent subsidence [13,22]. Despite these theoretical advantages, there is a paucity of clinical data regarding the use of small-sized stems in patients with exceptionally narrow femoral canals.

Comparing our results with the existing literature reveals several challenges.

Ko et al. reported on six hips (four patients) using extra-small stems (Bencox CM) in a cohort with a mean BMI of 25.7 kg/m^2^ and diagnoses including septic arthritis and juvenile rheumatoid arthritis. At a 2.3-year follow-up, their mean HHS improved to 88.8, though they reported two intraoperative fractures and one varus tilting [7]. In contrast, our cohort, primarily composed of DDH patients, achieved a higher mean HHS (96.4) and experienced no intraoperative femoral fractures, despite the technical complexity of achieving initial stability in these narrow anatomical structures.

Long-term studies of other specialized stems also provide context.

Drexler et al. reported a 19-year survival rate of 93.3% using 9 mm uncemented modular S-ROM stems in hypoplastic femurs [6]. Our patients had a comparable mean femoral canal diameter (8.5 mm vs. 9 mm) and mean weight (62.5 kg vs. 63 kg), yet our early survival rate remains 100%. However, it is imperative to note that our 100% survivorship reflects a short-to-midterm follow-up (mean 57 months). This cannot be directly compared to the 93.3% survival rate reported by Drexler et al., which accounts for a significantly longer 19-year period. The longer exposure to mechanical stress and polyethylene wear in long-term studies naturally increases the probability of late aseptic loosening, a factor that has not yet manifested in our cohort due to the limited time elapsed.

DiFazio et al. reported good clinical outcomes of 16 THA in 11 patients (mean height 152 cm) with dysplastic hips treated with the custom-made cemented swan-neck stem. The survival rate of this implant was 94%. The average follow-up period was 13.3 years [23].

Our group of patients is comparable; the mean height was 159 cm. The follow-up was shorter.

Similarly, Shahin et al. reported a 98.8% survival rate at 7.8 years using the conical Wagner stem, noting hypertrophic bone remodeling in 82 stems [24].

Regarding the Minima stem specifically, Drosos et al. reported 100% survivorship at 3 years in a series of 61 patients, although only 21.6% had DDH [13]. They observed reactive cortical hypertrophy in Gruen zones 3 and 5, consistent with our findings of bony trabecular development in these zones. This pattern of bone remodeling suggests a physiological load transfer through the proximal femur, a finding supported by biomechanical studies by Hert et al., which localized maximal cortical hypertrophy to Gruen zone 3 [15].

Tatani et al. and Tottas et al. published promising results of the Minima stem, compared with the other implants [21,22,25].

Christodoulou et al. also confirmed 100% survivorship of the Minima stem combined with ST-C cups at a 3-year follow-up, which aligns with our results [26].

In our cohort, we observed similar favorable survivorship with the ST-C cups; however, the present study focuses primarily on the femoral stem’s performance, as the clinical outcomes of these specific cups have been previously published [27].

### Limitations

This study has several limitations. First, the retrospective design and the small sample size without a control group limit the ability to draw definitive comparative conclusions. Second, we did not utilize dual-energy X-ray absorptiometry (DEXA) to quantify changes in bone mineral density, relying instead on conventional radiographic assessment. Third, the discrepancy in follow-up durations between our study and the established long-term literature (e.g., Drexler et al., Shahin et al.) must be emphasized. While our results are promising, they represent early clinical success and do not yet account for long-term complications such as late-onset instability or fatigue-related failure.

Finally, while the 57-month follow-up is sufficient for short-to-midterm evaluation, longer-term data are required to confirm the durability of these implants in younger, active patients.

## 5. Conclusions

Primary THA using the size 1 Minima stem in patients with narrow femoral canals (mean diameter 8.6 mm) provides promising clinical and radiological outcomes. The 100% survival rate and significant improvement in HHS at a mean follow-up of nearly five years suggest that this conservative stem is a reliable option for this challenging patient population.

## Figures and Tables

**Table 1:** Radiological changes aroud Minima stem in Gruen zones 1–7.

Zone	Bony Trabecular	Radiolucent Lines	Reactive Lines	Femoral Cortical
(Gruen)	Development	*n* (%)	*n* (%)	Hypertrophy
	*n* (%)			*n* (%)
1	0	0	0	0
2	0	1 (5.5%)	0	2 (11.1%)
3	5 (27.8%)	1 (5.5%)	2 (11.1%)	2 (11.1%)
4	1 (5.5%)	1 (5.5%)	4 (22.2%)	1 (5.5%)
5	3 (16.7%)	1 (5.5%)	2 (11.1%)	2 (11.1%)
6	0	1 (5.5%)	0	0
7	0	1 (5.5%)	0	0

## Data Availability

The original contributions presented in this study are included in the article. Further inquiries can be directed to the corresponding author.

## Abbreviations

The following abbreviations are used in this manuscript:

THA	Total hip arthroplasty
DDH	Developmental dysplasia of the hip
CCD	Caput–collum–diaphyseal angle
LLD	Limb length discrepancy
HHS	Harris Hip Score
SD	Standard deviation
BMI	Body mass index

## Appendix A

**Table A1:** Patients’ demographics and details of the group with the Minima stem size 1.

Parameters		Values		
No. of hip arthroplasties (unilateral-bilateral)		18/0		
Gender (male/female)		0/18		
Mean age (years, range, SD)		51.6 (24–71, SD 11.5)		
Mean follow-up (months, range, SD)		57 (37–77, SD 13.3)		
Mean BMI kg/m^2^ (range, SD)		24.8 (19.05–32.83, SD 3.8)		
Dorr classification (cases)				
	A	12		
	B	6		
	C	0		
Diagnosis of primary implantation (cases)				
	Postdysplastic osteoarthritis	13		
	Primary coxarthrosis	4		
	Avascular femoral head necrosis	1		
CCD angle (degrees, range, SD)				
	Preoperative	137.0 (116–153, SD 7.7)		
	Postoperative	135.0 (134–146, SD 2.9)		
LLD (mm, range, SD)				
	Preoperative	8 (0–50, SD 1.2)		
	Postoperative	0 (0–5, SD 0.2)		
Harris Hip Score				
	Mean preoperative (points, range, SD)	38.3 (24–56, SD 6.1)		
	Mean at final evaluation (points, range, SD)	96.4 (84–100, SD 4.2)		
